# *Neoceroplatus betaryiensis* nov. sp. (Diptera: Keroplatidae) is the first record of a bioluminescent fungus-gnat in South America

**DOI:** 10.1038/s41598-019-47753-w

**Published:** 2019-08-05

**Authors:** Rafaela L. Falaschi, Danilo T. Amaral, Isaias Santos, Adão H. R. Domingos, Grant A. Johnson, Ana G. S. Martins, Imran B. Viroomal, Sérgio L. Pompéia, Jeremy D. Mirza, Anderson G. Oliveira, Etelvino J. H. Bechara, Vadim R. Viviani, Cassius V. Stevani

**Affiliations:** 1 Departamento de Biologia Estrutural, Molecular e Genética, Programa de Pós-Graduação em Biologia Evolutiva, Universidade Estadual de Ponta Grossa, Ponta Grossa, PR Brazil; 2 Depto Física, Química e Matemática, Graduate School of Biotechnology and Environmental Monitoring (UFSCar), Sorocaba, SP Brazil; 3 IPBio - Instituto de Pesquisas da Biodiversidade, Iporanga, SP Brazil; 4 Departamento de Química, Instituto de Ciências Ambientais, Químicas e Farmacêuticas, Universidade Federal de São Paulo, Diadema, SP Brazil; 5 Departamento de Oceanografia Física, Química e Geológica, Instituto Oceanográfico, Universidade de São Paulo, São Paulo, Brazil; 6 Departamento de Química Fundamental, Instituto de Química, Universidade de São Paulo, São Paulo, Brazil

**Keywords:** Tropical ecology, Entomology, Photobiology

## Abstract

Blue shining fungus gnats (Diptera) had been long reported in the Waitomo caves of New Zealand (*Arachnocampa luminosa* Skuse), in stream banks of the American Appalachian Mountains (*Orfelia fultoni* Fisher) in 1939 and in true spore eating Eurasiatic *Keroplatus* Bosc species. This current report observes that similar blue light emitting gnat larvae also occur nearby the Betary river in the buffer zone of High Ribeira River State Park (PETAR) in the Atlantic Forest of Brazil, where the larvae were found when on fallen branches or trunks enveloped in their own secreted silk. The new species is named *Neoceroplatus betaryiensis* nov. sp. (Diptera: Keroplatidae: Keroplatinae: Keroplatini) based on a morphological analysis. *Neoceroplatus betaryiensis* nov. sp. larvae emit blue bioluminescence that can be seen from their last abdominal segment and from two photophores located laterally on the first thoracic segment. When touched, the larvae can actively stop its luminescence, which returns when it is no longer being agitated. The *in vitro* bioluminescence spectrum of *N*. *betaryiensis* nov. sp. peaks at 472 nm, and cross-reactivity of hot and cold extracts with the luciferin-luciferase from *Orfelia fultoni* indicate significant similarity in both enzyme and substrate of the two species, and that the bioluminescence system in the subfamily Keroplatinae is conserved.

## Introduction

The family Keroplatidae comprises of at least 92 genera and approximately 950 species^1–5^, distributed in all biogeographic regions. It is comprised of three subfamilies - Arachnocampinae, Macrocerinae, and Keroplatinae^6^, however Papp and Ševčik proposed a new subfamily^7^ named Sciarokeroplatinae based on a single species found the Oriental region. In the Neotropical region, there exist 32 genera and more than 200 species, of which 40 occur in Brazil^5,8^. Keroplatids inhabit mainly moist tropical forests usually associated with fungi. Adults can be collected in dark and humid places like stream banks and wet caves by sweeping in low vegetation, under hanging rocks or decaying logs. Usually, flies are caught by Malaise traps, UV lamp traps and occasionally in yellow pan traps^4^. Larvae are also present in moist and dark places, like caves, trunks, slits in stones, and have a variety of feeding habits. Predaceous larvae are seen in all subfamilies^9^, whereas mycophagy characterizes Keroplatinae^4,10^.

A few articles report on the function of bioluminescence of Diptera^11–13^. The larvae of luminous species such *Archnocampa luminosa* and *Orfelia fultoni* are carnivorous, however the Japanese *Keroplatus nipponicus* is fungivorous. Cannibalism is also common in the case of *O*. *fultoni*. Webs can be found in luminous and non-luminous mycetophilids. They are sticky and, in some cases, poisonous (*i*.*e*., contains oxalic acid as in the case of *O*. *fultoni*). Bigger arthropods such as cockroaches and ants were found caught in the webs of *O*. *fultoni*^14^. Sivinski performed experiments using transparent and blackened petri dishes covered with adhesive to verify whether light emission by *O*. *fultoni* can attract insects^11^. Most insects captured consisted of small Diptera (cecidomyids and phorids), which corroborated his hypothesis that preys were disoriented by the light emitted by the larva. In the case of a fungivorous species, like *K*. *nipponicus*, Sivinski hypothesized bioluminescence can perform other functions such as repelling negatively phototropic predators or as an aposematic signal^12^.

The subfamily Keroplatinae holds the highest number of genera (70) and species (677), in which it is possible to recognize two distinct tribes according to Matile^2,15^: Keroplatini and Orfeliini. The tribe Keroplatini contains 153 species in 21 genera^16^, which are characterized mainly by short, two-segmented palpi and laterally compressed or otherwise modified antennae. Of the few known immature specimens, the larva always has four anal lobes. Bioluminescence in Diptera is reported only in the Keroplatidae family in the genera Arachnocampa Edwards (Arachnocampinae), Keroplatus Bosc (Keroplatinae: Keroplatini), and Orfelia Costa (Keroplatinae: Orfeliini)^4,16,17^. Light emission by a species of Mallochinus Edwards of the Keroplatini group is uncertain^18^. Yet, according to Lloyd^19^ an unknown luminous species of Keroplatidae was found in New Guinea.

Notably, within the Keroplatidae there are at least two morphologically and biochemically distinct bioluminescent systems, namely those of Arachnocampa and *O*. *fultoni* Fisher^20^, whilst the bioluminescent system of Keroplatus species remains unknown. In Arachnocampa larvae, bioluminescence is produced by a lantern at the tip of the abdomen which is derived from Malpighian tubules^20^, and involves an ATP-dependent luciferase as in the case of beetle luciferases^21^. The chemical structure of the Arachnocampa luciferin was shown to derive from xanthurenic acid and tyrosine but has not yet been elucidated^22^. On the other hand, the bioluminescence system of *O*. *fultoni*, produced by translucent areas associated with rows of black bodies, involves an unknown heterodimeric 140 kDa luciferase, an unknown luciferin and a Substrate Binding Fraction (SBF), which apparently releases luciferin in the presence of reducing agents^20^. Notably, there is no cross-reaction between either the luciferin or the luciferase of Arachnocampa and Orfelia.

The genus Neoceroplatus Edwards is represented by twelve species worldwide, of which only *N*. *samiri* Khalaf is from the Neartic region. The other eleven species reportedly occur in the Neotropics^4^. Seven *Neoceroplatus* species are known: *N*. *hodeberti* Matile, *N*. *lauroi* Lane, *N*. *punctipes* Matile, *N*. *minimax* Edwards, *N*. *dissimilis* Matile, *N*. *paicoenai* Lane and *N*. *spinosus* Matile; the last four ones found in the State of São Paulo. Matile offers a taxonomic key for all these Neotropical species^2^. In this work, a new species of Keroplatini is described occurring in a conservation area of Atlantic Forest in São Paulo State named Betary Reserve, which is the first record of blue bioluminescent species of Diptera in the Neotropical region. Additionally, this work increases the number of known *Neoceroplatus* species to thirteen. Importantly, its luciferin and luciferase seem to be similar to the ones present in *O*. *fultoni* as attested by cross-reaction assays between both species.

## Results and Discussion

### Observation of larval behavior and bioluminescence

**Figure 1:**
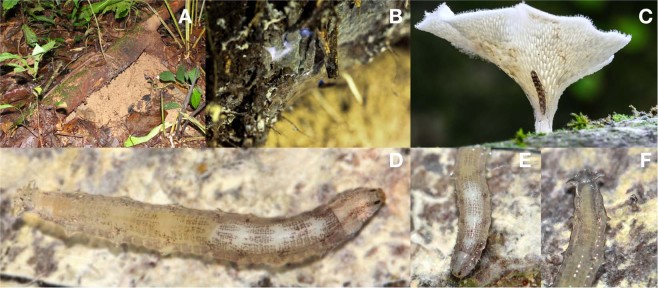
Different locations and habitats where larvae of *Neoceroplatus betaryiensis* nov. sp. were photographed. (**A**) Decaying log where larvae were collected. (**B**) Larvae on the surface of the log surrounded by a web-like mucus. (**C**) Association of a larva with a *Favolus brasiliensis* (Fr.) Fr. mushroom raised in a terrarium. (**D**) Photo of a typically translucid *N*. *betaryiensis* sp. nov. (**E**) Details of the larva head and (**F**) last abdominal segment.

**Figure 2:**
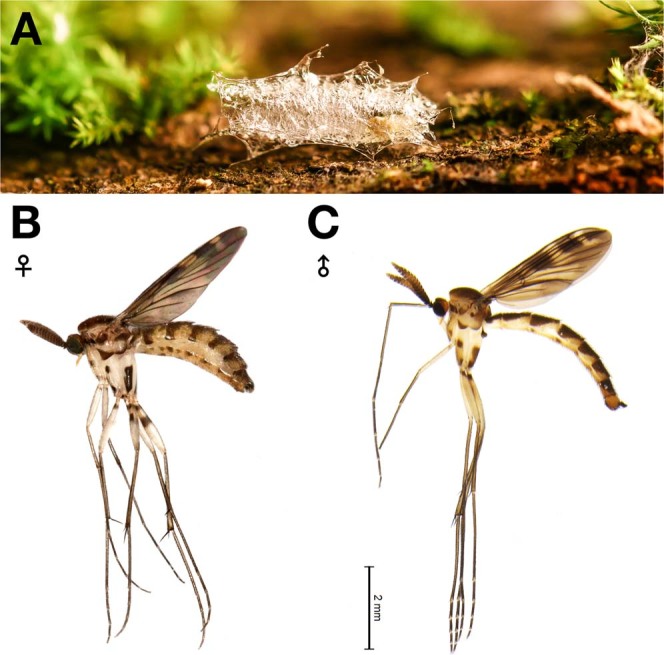
Life cycle of *Neoceroplatus betaryiensis* nov. sp. (**A**) Pupal stage. (**B**) Emerged adult female. (**C**) Emerged adult male.

Larvae are usually found on fallen branches or tree trunks, where they are lodged between the wood and surrounded by their secreted mucus. They can also be found on tree trunks about a meter above the ground (Fig. 1). Typically, two or three larvae can share a single branch, although as many as 15 have once been collected from a single branch. Some larvae were also spotted in association with an unidentified species of brownish polypore mycelium. The larvae are very active, especially at night and can move constantly whilst completely covered by mucus. When disturbed, they quickly move under their mucus. Before pupation, they construct a cocoon on the surface of the log beneath either moss or fungi, where the pupae stay until the adult emerges (Fig. 2). Pupae are also bioluminescent when observed using a CCD camera (image not presented).

**Figure 3:**
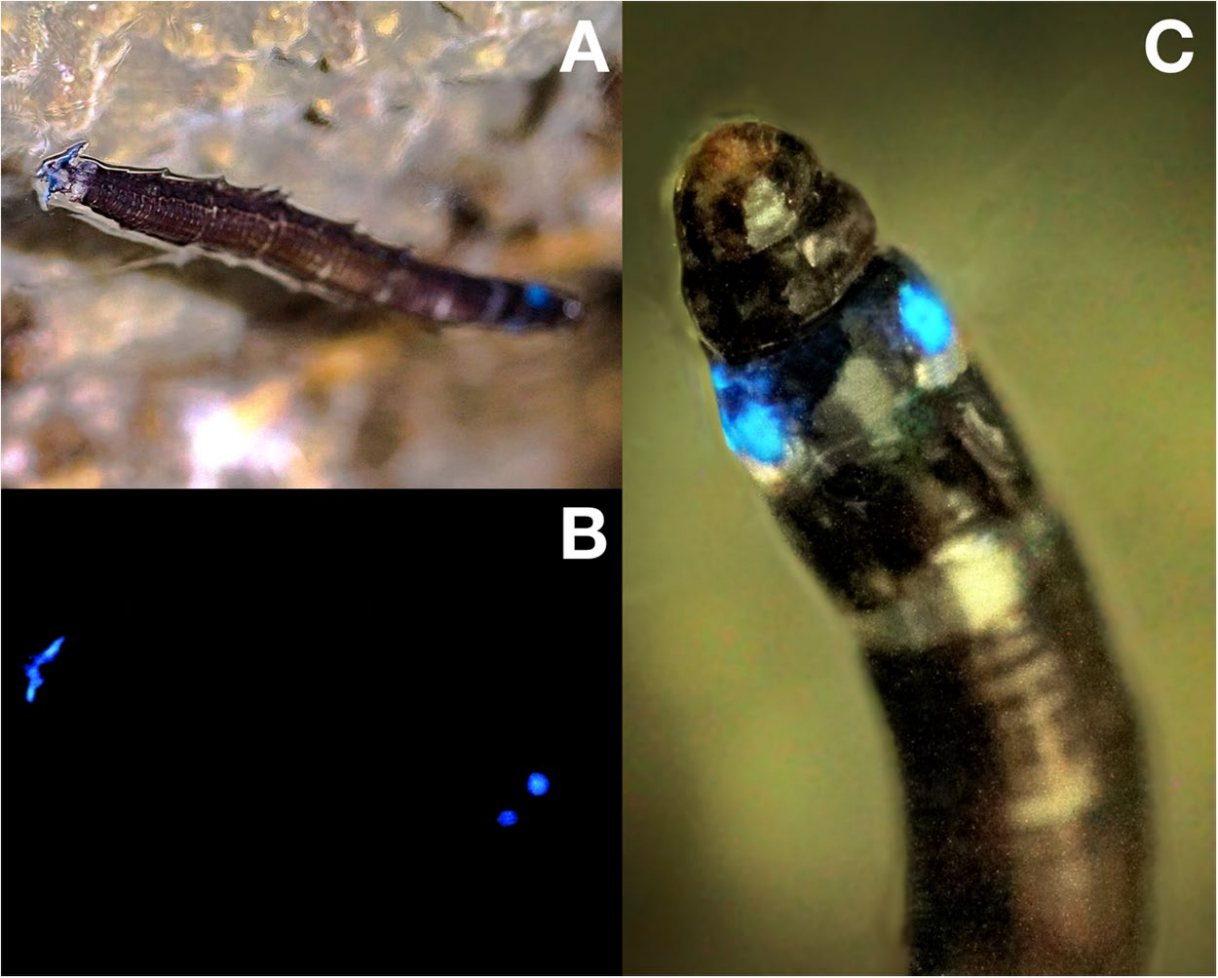
Bioluminescence of *Neoceroplatus betaryiensis* nov. sp. larvae. (**A**) Light emission under illumination and (**B**) in the dark. (**C**) Detailed view of the two photophores located laterally on the first thoracic segment.

*Neoceroplatus betaryiensis* nov. sp. larvae emit blue light from their last abdominal segment and from two photophores located laterally on the first thoracic segment (Fig. 3). Until now, it was not possible to ascertain whether the bioluminescence is associated to the black bodies as in the case *Ofelia fultoni* and possibly of *Keroplatus* spp., but with the presence of several dark granules spread along the body suggest this is a possibility. Light emission is turned off when the larva is touched and begins again a few minutes after this mechanical agitation ceases. We were not able to observe the eating habits of the larvae, although the absence of remnants of insects trapped in the web with the proximity of fungi suggest that the larvae could be fungivorous.

**Figure 4:**
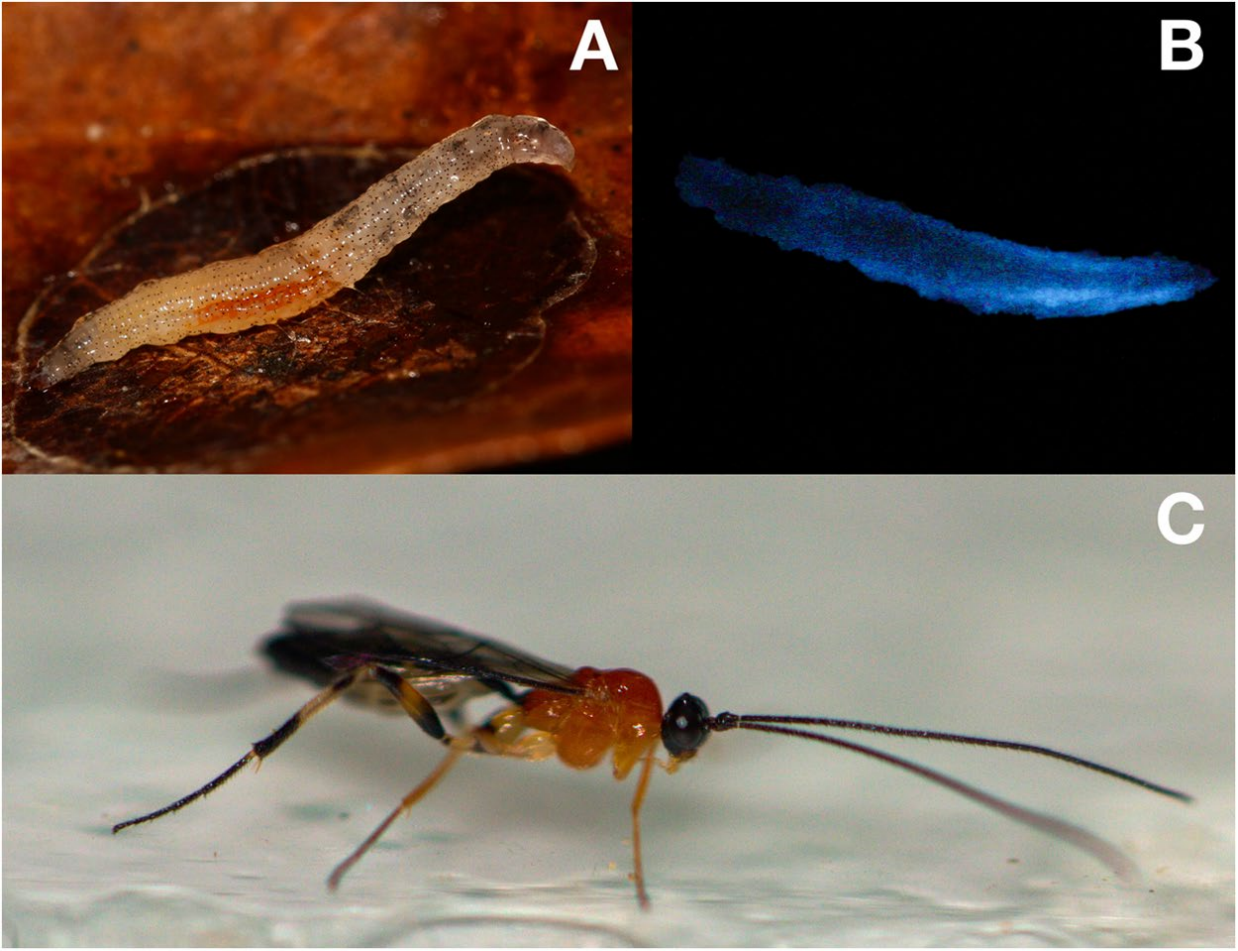
Parasitized unidentified luminous dipteran larva. (**A**) Larva under illumination and (**B**) light emission from the entire body. (**C**) Ichneumonid parasitic wasp that emerged from the pupa.

Unexpectedly, a different larva was once pulled out from the underside of a fallen leaf, showing diffuse blue bioluminescence apparently consisting of small brown photophores along the whole body (Fig. 4A,B). It exhibited bizarre behavior compared to other larvae observed, as it moved slower and exposed itself more than was previously observed. At first, it was thought to be another luminous dipteran species. However, when kept in a terrarium, the larva entered the pupal stage two weeks later and after eleven days, an ichneumonid parasitic wasp emerged from the pupa (Fig. 4C). Luminous mycetophilids are reportedly attacked by hymenopterous parasitoids^12^ and this diffuse bioluminescence could be the result of either a defensive reaction against the parasite or the consequence of internal organ damage spreading the photogenic material along the body of the larva. However, it may also belong to another new species that can emit light along the whole body as observed in *Keroplatus nipponicus*^13^.

### Chemiluminescence and luciferin-luciferase cross-reaction

**Figure 5:**
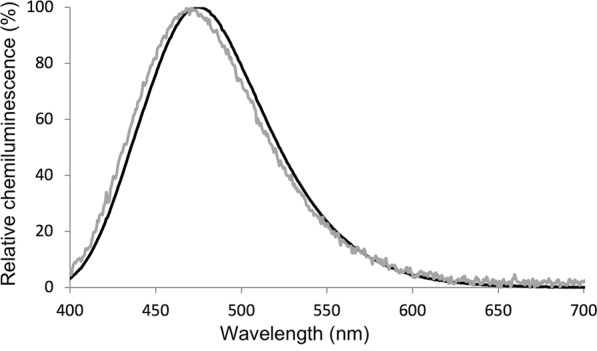
Chemiluminescence spectrum obtained from the reaction of luciferase and hot extracts of *Orfelia fultoni* (gray) and *Neoceroplatus betaryiensis* nov. sp. cold extract plus *O*. *fultoni* hot extract (black).

Whole *Neoceroplatus betaryiensis* nov. sp. larvae were homogenized in the extraction buffer. The resulting homogenate displayed considerable light emission. After centrifugation a weaker luminescence remained in the supernatant, indicating that the luciferin and luciferase are active and solubilized in these conditions. The supernatant or “cold extract” showed a gradual increase in light emission to a higher intensity upon the addition of DTT (dithiothreitol) as a reducing agent. This is similar to what occurs with crude extracts of *Orfelia*, consistent with the presence of the Substrate Binding Fraction (SBF) which retains luciferin. Addition of *O*. *fultoni* hot extract that contains the luciferin to *Neoceroplatus* cold extract (luciferase-rich fraction) also increased light emission. The spectrum obtained by cross-reacting the cold extract of *N*. *betaryiensis* nov. sp. and the hot extract of *O*. *fultoni* in the presence of DTT shows a maximum intensity of around 472 nm (Fig. 5), which matches the recently reported value for *O*. *fultoni* luciferin-luciferase system^23^. The addition of hot extract of *N*. *betaryiensis* nov. sp. to the purified luciferase of *O*. *fultoni* also resulted in blue light emission. These results indicate that *N*. *betaryiensis* nov. sp. and *O*. *fultoni* share either very similar or identical luciferin substrates and luciferase enzymes.

### Molecular phylogenetic studies

**Figure 6:**
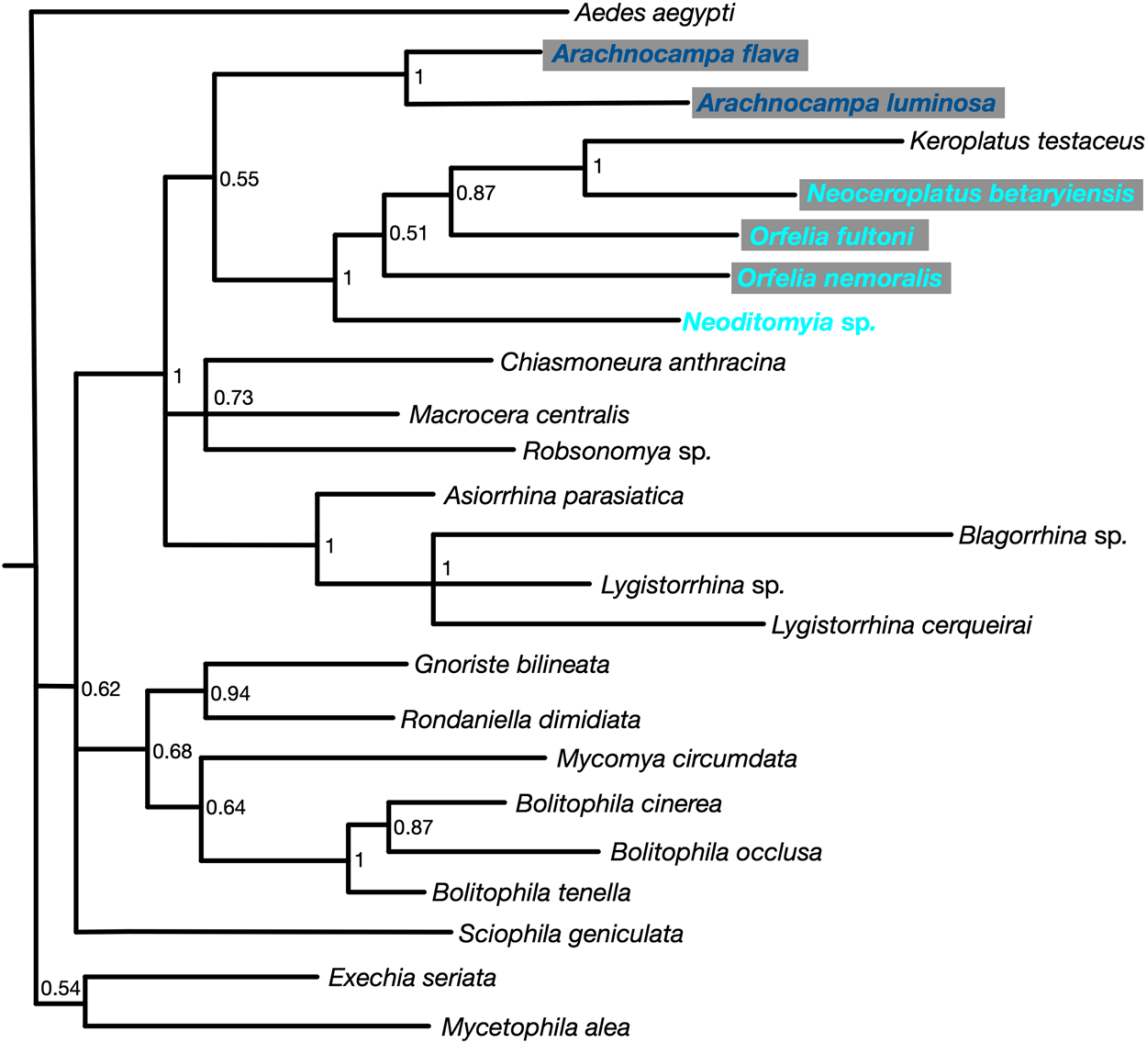
Phylogenetic tree of Keroplatidae bioluminescent and non-bioluminescent species using mitochondrial gene cytochrome oxidase I (COI). Species surrounded by a grey rectangle are bioluminescent. Species whose name is displayed in pale blue share the same specific luciferin and SBF and the species with name in dark blue, share the same specific luciferase and luciferin.

A molecular phylogenetic analysis was conducted with *N*. *betaryiensis*, *Orfelia fultoni* and the non-luminescent *Neoditomiya* sp., using the mitochondrial gene of cytochrome oxidase I (COI). COI barcode-gene is useful to recognize closely related species, to investigate their relative phylogenetic position, and to analyze if there was an evolutionary relationship among the luciferin-luciferase systems of Keroplatinae. The molecular phylogenetic analysis showed that the fungus-gnat *N*. *betaryiensis* nov. sp. is positioned as a sister-group of the luminescent *Keroplatus* genus (Fig. 6), followed by the non-luminescent *Neoditomyia* (Lane & Sturm) and the luminescent *O*. *fultoni*, which is in agreement with the literature^2,10^. The placement of the non-bioluminescent *Neoditomyia* sp. as a sister-group of Keroplatinae subfamily was also observed. Interestingly, *Orfelia*-type luciferin and its Substrate Binding Protein (SBF) has been recently reported in *Neoditomyia* sp.23. Both the luciferin and SBF of *Neoditomyia* were able to cross-react with purified *O*. *fultoni* luciferase, resulting in an emission spectrum that overlaps with that of *O*. *fultoni* extracts. The cross-reactions between *O*. *fultoni* and *N*. *betaryiensis* nov. sp. strongly suggest that the same bioluminescent system is also shared by these genera. Altogether, the COI gene analysis and biochemical results indicate that *Neoditomyia* spp., *Neoceroplatus betaryiensis* and *Orfelia fultoni*, and by inference *Keroplatus* species, share the same bioluminescent system.

### Taxonomical description

*Neoceroplatus betaryiensis* Falaschi, Johnson & Stevani nov. sp. (Figs 1B to 4B, S1A to S5D).

Material examined. **Holotype**: Male, BRAZIL, São Paulo, Iporanga, Reserva Betary, IPBio – Instituto de Pesquisas da Biodiversidade, 24°35′27″S 48°37′44″W, 120 m, manual collection on the underside of a leaf (on June 14th 2017 one larva was collected and on August 24th 2017 the adult male emerged), Domingos, A. H. R., Santos, I. & Johnson, G. A. cols. [MZUSP- MZ052800] (specimen pinned with terminalia on permanent slide). **Paratypes**: Two females, same data as holotype, except 01.v.2017 (larva collected) 24.v.2017 (adult female emerged) [MZUSP-MZ052801] (specimen pinned), [MZUSP-MZ052802 (specimen on permanent slide); one larva, same data as holotype, except 15.v.2017, [MZUSP-MZ052803] (in 80% ethanol); two larvae, same data as holotype, except 16.iv.2017, [MZUSP-MZ052804] (in 80% ethanol), [MZUSP-MZ052805] (in permanent slide); pupa exuvium, same data as holotype, except 24.viii.2017, [MZUSP-MZ052806] (in permanent slide); net remains, same data as holotype, except 24.viii.2017, [MZUSP-MZ052807] (in 80% ethanol).

**Etymology**: The specific epithet refers to the Betary brook, in whose banks the specimens were collected.

### Diagnosis and comments

*Neoceroplatus betaryiensis* nov. sp. can be distinguished from the other Neotropical *Neoceroplatus*¸ especially from *N*. *dissimilis*, its closest species, by the shape of the genitalia, particularly the gonostylus (Figs S3E, S4B,C) and the absence of spines in the gonostylus as appears in *N*. *paicoenai*.

#### Male

Body length (without antennae): 5.2 mm (Fig. 2C). Wing length: 3.75 mm (Fig. S1F). Terminalia length: 0.28 mm (Figs S3A,C,E; S4A,B). *Head:* Brownish. Three ocelli in triangular position, each positioned on a callus. The lateral ocellus larger than middle one (Fig. S1D). The distance between the lateral ocelli two times its own diameter, separated from the edge of the eye at a distance slightly higher than its own diameter. Median ocellus approximately 3/4 smaller than lateral ocellus. Compound eyes are large, about 1.5 times higher than wide in lateral view, covered with microsetae. Antenna (Fig. S1A,B) three times higher than head, flattened laterally, yellowish-brown. Scape and pedicel cylindrical, both slightly wider than high, brownish. Flagellum expanded and flattened, with 14 flagellomeres, dorsal and ventral macrosetae covering flagellomeres. Terminal flagellomere, longer than wide, with a minute, thin yellow-whitish apical process at the apex, in a shallow notch (Fig. S1B). Labrum and labella weakly sclerotized; labellum exceeding the ventral edge of the eyes. Palpus bi-segmented. Second palpomere elongated, covered with setae laterally, pointed at the apex, as long as face and clypeus combined length; medially membranous, weakly sclerotized. *Thorax*: Mostly yellowish, with brownish spots laterally. Antepronotum brownish, covered with macrosetae; proepisternum brownish, bare; anterior spiracle surrounded by setae; anepisternum bare, apically yellowish and brownish at the base and internal edge; katepisternum and anepimerum bare, mostly yellowish, with a brownish spot proximally; laterotergite mostly brownish, covered with long and sparse macrosetae. Scutum brownish, densely covered with macrosetae. Scutellum brownish, covered with long macrosetae. Mediotergite bare, mostly brownish. Halter with brownish knob and stem mostly yellowish, with a row of setae. *Wing*: (Fig. S1F) Wing membrane infuscate brown to dark brown mainly on distal half, basal cells yellowish with small brown maculae; the minor hyaline area between R_4_ and R_5_, and C to M_1_. Microsetae irregularly scattered throughout wing. Veins brown to dark brown. Strong setation on C, R_1_, R_4_ and R_5_. Humeral lightly shorter than half of R_1_. Vein C exceeding apex of R_5_, at a distance beyond R_5_ that is roughly 1/4 the distance between R_5_ and M_1_ apex. R_4_ ending at R_1_ apex, bending posteriorly with almost right angle with R_5_. M_1_, M_2_, M_4_ and anal veins not reaching the wing margin. A_1_ as long as CuP vein. *Legs*: Mostly white to yellowish. Fore coxa white-yellowish with brownish spot and covered with macrosetae on its all anterolateral face. Mid coxa white-yellowish with two brownish spots covered with macrosetae anterolaterally; hind coxa white-yellowish with a large brownish spot covered with macrosetae only laterally. Femora white-yellowish, covered with setae, with an upper brownish spot antero-laterally. Tibiae and corresponding tarsi yellowish to pale brown, covered with regular rows of setae. *Abdomen*: Covered with macrosetae. Mostly yellowish ventrally and laterally, with large brownish areas dorsally. Sternites 3–6 with two brownish marks in each lateral margin (Fig. S2A–C). *Terminalia*: Mostly yellow (Fig. S3A,C,E) covered with long, dense macrosetae (Fig. S4A,B). Tergite 9 yellow-brownish, V-shaped, longer than wide (Figs S3C,G, S4A). Cerci yellow, poorly sclerotized, covered with dark setae (Figs S3C,G, S4A). Gonocoxites partially fused basally, yellowish, almost entirely covered with long and dark setae, and few short and dark setae (Fig. S3A,E). Gonostylus dark brown, prominent, shark fin-shaped, covered with long setae, and with dark, thicker bristles on inner side (Figs S3E, S4B,C), with an interiorly-directed, antero-dorsal lobe (Fig. S3E).

#### Female

Body length (without antennae): 5.9 mm (Fig. 2B). Wing length: 4 mm (Fig. S1G). Terminalia length: 0.39 mm (Figs S3B,D,F; S4D–E). As the male, except as follows. *Head*: Last flagellomere slightly wider than long, subquadrate (Fig. S1C). *Wing*: the minor hyaline area between R_4_ and R_5_, and C to M_2_ and the major hyaline area reaching the apex of CuA. Humeral roughly more than half of R_1_. Vein C at a distance beyond R_5_ that is more than one third the distance between R_5_ and M_1_. R_4_ divergent relative to R_5_ forming a 45°. Base of M_4_ weakly sclerotized. A_1_ longer than CuP vein (Fig. S1G). *Abdomen*: (Fig. S2D,E) Dorsally yellowish with two large brownish spots on tergites 2–7. Sternites 2–3 white-yellowish with one subtriangular brownish mark and rounded shaped on 4–5. *Legs*: Mid coxa whitish with two dark-brownish spots covered with macrosetae antero-laterally, and a row with four macrosetae at the lateral apex; hind coxa whitish with a large dark-brownish spot covered with macrosetae laterally (Fig. S1E). Femora whitish, the hind femora with two brownish spots antero-laterally. *Terminalia*: mostly brownish (Figs S3B,D,F; S4D,E) covered with long, dense macrosetae, without microsetae. Sternite 8 covered with dense setae, inner margin straight; tergite 8 covered with setae; tergite 10 well developed; cercus formed by one article, entirely covered with setae (Fig. S4D,E).

#### Immature

Body length: 20.1 mm (Figs 1D, 3A, 4A and S5C); Pupa exuvium: 6,95 mm (Fig. S5D). Overall morphology similar to other Keroplatini larvae. General color gray-whitish, head capsule brown, well sclerotized, subquadrate (Fig. S5A,B). No distinctive modification of the body cuticle, except anterior portion of body divided into four subquadrate areas, followed by narrow transverse lines towards posterior end (Figs 1D–F, 3A, 4A and S5C), giving a segmented texture to the integument. Cephalic capsule quadrangular, slightly longer than wide, very short in relation to body width (Figs 3C, S5A,B); a well-developed gena, ventral part of foramen magnum longer than wide. Antenna very reduced, flattened, ellipse-shaped, protruding above the antenna base, positioned more dorsally. Labrum in continuity with the clypeal area. Premandible with row of elongated, flexible teeth, supported by a pair of lateral chitinous arms. Mandible semicircular and bearing two rows of medially directed teeth; Mandible well developed. Maxilla elongated, rather parallel distally, with fifteen teeth on inner border. Cardo slender, transverse (Fig. S5A). Secondary annulation on abdominal segments. Posterior end with a pair of lobose triangular projections (Figs 1D,F and 3A).

#### Comments

The anterior photophores are located on the first thoracic segment of the larva and the posterior on the last abdominal segments, without visible external morphological structures.

In the holotype and paratypes of *Chetoneura shennonggongensis* (Amorim & Niu) from the Zoology Museum of University of São Paulo, Brazil^24^, there are no structures in the last segment similar to the “posterior papillae”, described by Matile from the genera *Arachnocampa*, *Keroplatus*, and *Macrocera* in either *C*. *shennonggongensis* or *Neoceroplatus betaryiensis*. These structures can be properly described and understood from deeper histological studies, which was not the aim of this study.

## Conclusion

Here we report the discovery of the first bioluminescent species of fungus-gnats of the family Keroplatidae in the Neotropical region. Similar to the Palearctic and Oriental *Keroplatus* species, *N*. *betaryiensis* also lives under dead logs and is probably sporophagous. The bioluminescence is blue, and likely shares the same luciferin-luciferase system of the North-American *Orfelia fultoni*, and possibly of the Palearctic *Keroplatus* spp. These findings show how Neotropical biodiversity is still poorly known^25^, despite being recognized as the most diverse biogeographic region on the planet. Unfortunately, the anthropic pressure on natural areas has been increasing, causing disturbances in different habitats, with damage in megadiverse countries such as Brazil^26^. These threats affect especially small invertebrates, which have been extinguished at a much faster rate than their discovery and description^27^. This reinforces the need for conservation policies for areas such as the Betary Reserve, a place that provides new taxa for science, the Keroplatidae being one of them.

## Methods

### Diptera larva collection

The material presented here was collected by G.A.J., A.H.R.D. and I.S. on the property of the non-governmental organization Instituto de Pesquisas da Biodiversidade (IPBio), municipality of Iporanga, São Paulo State, Brazil. The Betary Reserve, the first branch of IPBio, is located between the geographical coordinates 24°35′16″S; 48°37′44″W, a preserved area of *ca*. 60 hectares of dense ombrophylous forest in an advanced regeneration status. The reserve is situated in the largest remaining continuous area of the Atlantic Forest, and within the protective zone of the Touristic State Park of High Ribeira River (PETAR). The fungus gnats were collected during a hot and rainy period, with relative humidity of 90%, and manually collected on fallen trees.

On May 1^st^ 2017, four larvae were collected and kept in terraria, and on May 24^th^ two female adults emerged. Another larva, with erratic behavior, collected on May 31, 2017 was parasitized by an ichneumonid wasp, which emerged on June 13^th^. In the laboratory the larvae were kept in large glass terraria with a thin fabric cover. The bottom of the terraria were covered with dried leaves, with branches leaning against the lateral glass walls, and with thicker branches that harbored mushrooms of the species *Favolus brasiliensis* (Fr.) Fr. It was observed that after a few days the larvae migrated to the bottom of the mushrooms, where they built their web. After a few days they went down to the leaves and about two weeks later they emerged as adults.

The female adults that emerged in the laboratory were primarily preserved in 70% ethanol. The larvae, which were preserved in KAAD solution (100 mL kerosene, 700 mL of absolute alcohol, 100 mL of acetic acid and 100 mL of colorless house detergent), were collected in the field. The larva remained in solution for 12 h and then 70% alcohol for 24 h. After that, the larva was removed and kept in 80% alcohol. Hot water prevented dryness and rapid “wrinkling” and KAAD preserved color.

### Larvae and adult preservation and microscopic examination

The material examined in this study was deposited in the Diptera collection at the Museu de Zoologia da Universidade de São Paulo (MZUSP, São Paulo, Brazil). The collected material was initially preserved in 80% ethanol. The terminalia were detached from the abdomen, cleared in 10% KOH aqueous solution at 40 °C for 40–60 min and promptly washed first in glacial acetic acid and then in 80% ethanol for 15 min. Each specimen and wings were soaked for 15 min twice both in absolute ethanol and then in xylenes (mixture of *o*,*m*,*p*-xylene) Synth, 98.5%. Afterwards, each specimen was mounted in the permanent slide with Canadian balsam. Wing and terminalia were drawn after mounting on permanent slides. Photographs were taken with using a Leica DC camera either attached to a Leica MZ16 stereomicroscope or to a Leica DM2500 microscope. Stacking was performed with Helicon Focus 6 and edited with the Adobe Photoshop CC 2017. General illustrations of male and female terminalia were drawn with the help of a camera lucida attached to the microscope, vectorized using Adobe Illustrator CC 2017. The species was identified using the available identification key present in Matile^2^ and by comparison with the types housed at the MZUSP (with the closest species – *Neoceroplatus dissimilis* and the other with the type specimens available: *N*. *dureti*; *N*. *hodeberti*; *N*. *lauroi*; *N*. *monostylus*; *N*. *paicoenai* and *N*. *spinosus*). The morphological terminology used here follows the literature^2,28^.

### Bioluminescence imaging

Imaging of bioluminescent larvae and pupae were done using a LightCapture II CCD camera (ATTO, Tokyo, Japan).

### Chemiluminescence assay

Between 3 to 6 entire *Neoceroplatus betaryiensis* nov. sp. larvae were homogenized in 1 ml of *Orfelia fultoni* extraction buffer (0.10 M phosphate buffer, 1 mM EDTA, 1% Triton X-100, pH 7.0). Hot-cold extract assays were performed using a previously established method for *O*. *fultoni*^20,23^. The homogenate of the larvae was separated in two aliquots, one of which was centrifuged and the pellet discarded. The supernatant was left to react completely, with the remaining solution being termed the “*cold extract*”. The second aliquot was treated with 10 mM DTT and was heated at 98 °C for 5 minutes in an anoxic atmosphere. This solution was then centrifuged and the pellet discarded, leaving the “hot extract”. Purified *O*. *fultoni* luciferase and luciferin containing hot extracts were prepared using larvae collected in North Carolina (USA) by one of the authors (VRV). Light emission was measured in counts per second (cps) using an ATTO AB2200 luminometer (Tokyo, Japan). Chemiluminescence spectra was recorded using an Atto LumiSpectra spectroluminometer (Tokyo, Japan).

### Molecular phylogenetic analysis

The phylogenetic analysis of Keroplatidae species was performed using the mitochondrial gene cytochrome oxidase I (COI). The nucleotide sequences were aligned using ClustalW algorithm^29^ on MEGA 6.0 software^30^. The jModelTest2 program^31^ was used to predict the best evolutionary model, which resulted in GTR + G + I substitution model. The phylogenetic analysis used by the software MrBayes 3.2^32^, through two separately runs with 10,000,000 generations each. The first 25% of trees were discarded and concatenated to create a *consensus* tree.

## Supplementary information

Video S1

Video S2

Supplementary Information
