# Supplementary material for: Neoceroplatus betaryiensis nov. sp. (Diptera: Keroplatidae) is the first record of a bioluminescent fungus-gnat in South America
Source: Sci Rep. 2019 Aug 5;9:11291. doi: 10.1038/s41598-019-47753-w (PMC6683459; doi:10.1038/s41598-019-47753-w)
Supplement: Supplementary file 3 — Supplementary Information [file 41598_2019_47753_MOESM3_ESM.pdf]

SUPPLEMENTARY DATA

Neoceroplatus betaryiensis nov. sp. (Diptera: Keroplatidae) is the first record of a bioluminescent fungus-gnat in South America

Rafaela L. Falaschi[1], Danilo T. Amaral[2], Isaias Santos[3], Adão H. R. Domingos[3], Grant A. Johnson[3], Ana G. S. Martins[3], Imran B. Viroomal[3], Sérgio L. Pompéia[3], Jeremy D. Mirza[4,5], Anderson G. Oliveira[5], Etelvino J. H. Bechara[6,*], Vadim R. Viviani[2,*] & Cassius V. Stevani[6,*]

[1]Departamento de Biologia Estrutural, Molecular e Genética, Programa de Pós-Graduação em Biologia Evolutiva, Universidade Estadual de Ponta Grossa, Ponta Grossa, PR, Brazil. [2]Depto Física, Química e Matemática, Graduate School of Biotechnology and Environmental Monitoring (UFSCar), Sorocaba, SP, Brazil. [3]IPBio - Instituto de Pesquisas da Biodiversidade, Iporanga, SP, Brazil. [4]Departamento de Química, Instituto de Ciências Ambientais, Químicas e Farmacêuticas, Universidade Federal de São Paulo, Diadema, SP, Brazil. [5]Departamento de Oceanografia Física, Química e Geológica, Instituto Oceanográfico, Universidade de São Paulo, Brazil. [6]Departamento de Química Fundamental, Instituto de Química, Universidade de São Paulo, São Paulo, Brazil. Correspondence and requests for materials should be addressed to V.R.V (email: viviani@ufscar.br), E.J.H.B. (email: ebechara@iq.usp.br) or C.V.S (email: stevani@iq.usp.br)

TABLE OF CONTENTS

Figure S1. *Neoceroplatus betaryiensis* nov. sp. (**A**) Male antenna. (**B**) Male last flagellomere in detail. (**C**) Female antenna. (**D**) Female dorsal view of the thorax. (**E**) Female lateral view of the thorax. (**F**) Male wing (**G**) Female wing.

Figure S2. *Neoceroplatus betaryiensis* nov. sp. (**A**) Male dorsal view of the abdomen. (**B**) Male ventral view of the abdomen. (**C**) Male lateral view of the abdomen. (**D**) Female dorsal view of the abdomen. (**E**) Female ventral view of the abdomen. (**F**) Female lateral view of the abdomen. Scale bar: 1 mm.

Figure S3. *Neoceroplatus betaryiensis* nov. sp. (**A**) Ventral view of the male terminalia. (**B**) Dorsal view of the female terminalia. (**C**) Dorsal view of the male terminalia. (**D**) Ventral view of the female terminalia. (**E**) Lateral view of the male terminalia. (**F**) Lateral view of the female terminalia.

Figure S4. *Neoceroplatus betaryiensis* nov. sp. (**A**) Dorsal view of the male terminalia. (**B**) Lateral view of the male terminalia. (**C**) Detail of the inner face of the gonostylus. (**D**) Dorsal view of the female terminalia. (**E**) Lateral view of the female terminalia. Abbreviations: Aed, aedeagus; Cs, cercus; Gs, gonostylus; Gx, gonocoxite; S8, sternite 8; T10, tergite 10; T8, tergite 8; T9, tergite 9.

Figure S5. *Neoceroplatus betaryiensis* nov. sp. (**A**) Larva, ventral view of the head. (**B**) Larva, dorsal view of the head. (**C**) Larva, dorsal view of the body (divided into four subquadrate areas). (**D**) Pupa exuvium.

Video S1. Larva of *Neoceroplatus betaryiensis* nov. sp. walking on a mushroom of *Favolus brasiliensis*.

Video S2. Larva of *Neoceroplatus betaryiensis* nov. sp. building up its web on a leaf.

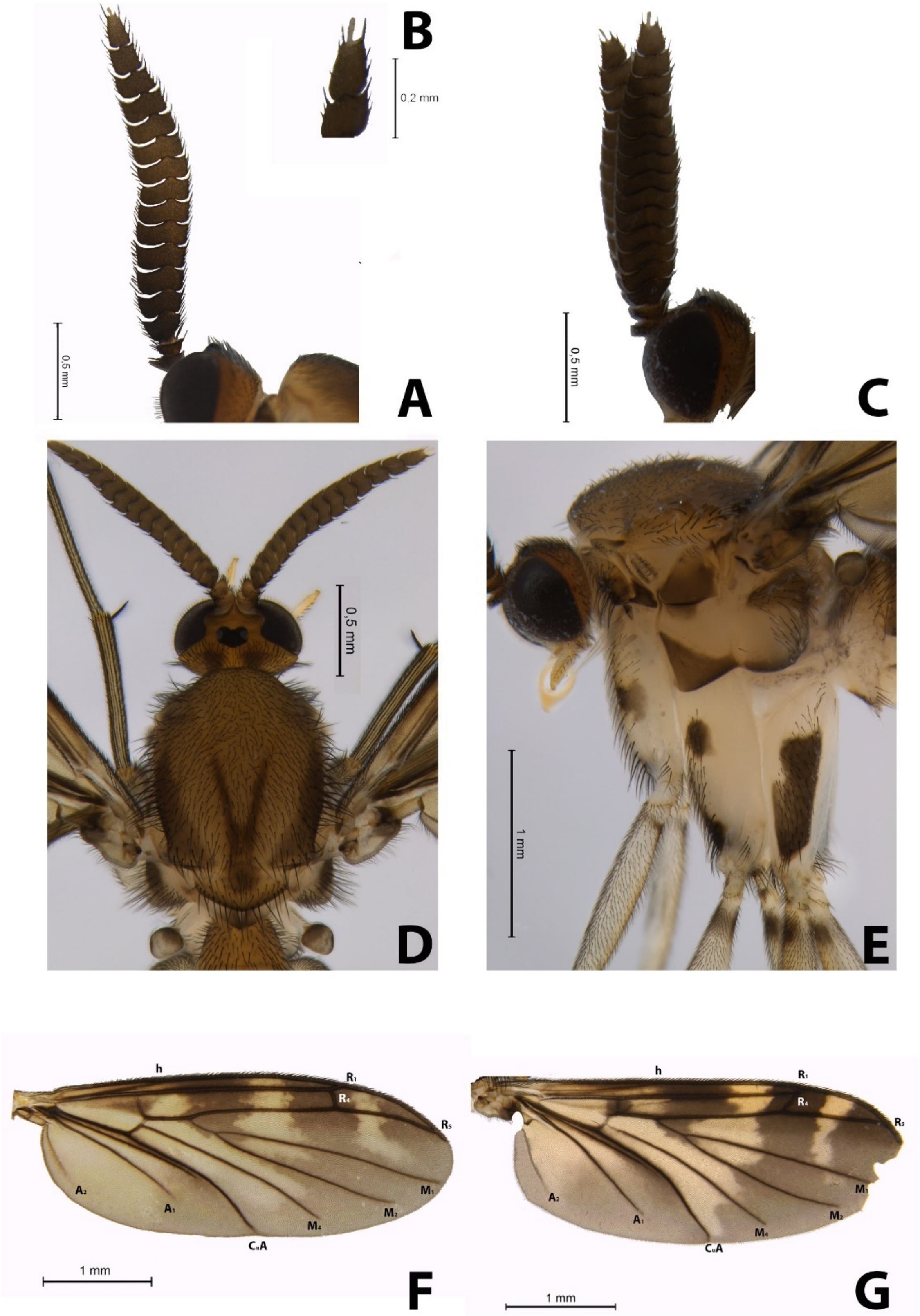

Figure S1. *Neoceroplatus betaryiensis* nov. sp. (**A**) Male antenna. (**B**) Male last flagellomere in detail. (**C**) Female antenna. (**D**) Female dorsal view of the thorax. (**E**) Female lateral view of the thorax.

(**F**) Male wing (**G**) Female wing.

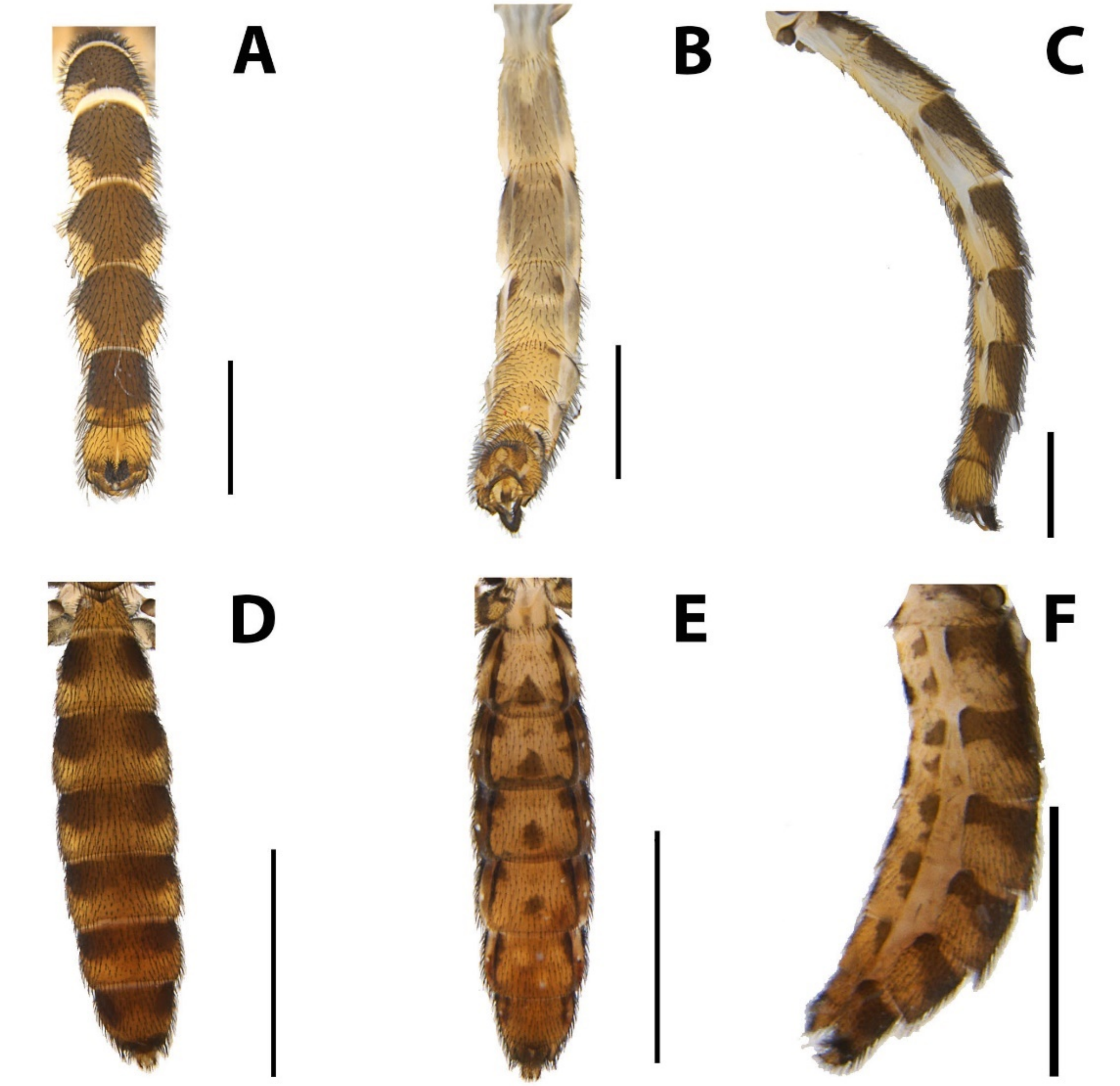

Figure S2. *Neoceroplatus betaryiensis* nov. sp. (**A**) Male dorsal view of the abdomen. (**B**) Male ventral view of the abdomen. (**C**) Male lateral view of the abdomen. (**D**) Female dorsal view of the abdomen. (**E**) Female ventral view of the abdomen. (**F**) Female lateral view of the abdomen. Scale bar: 1 mm.

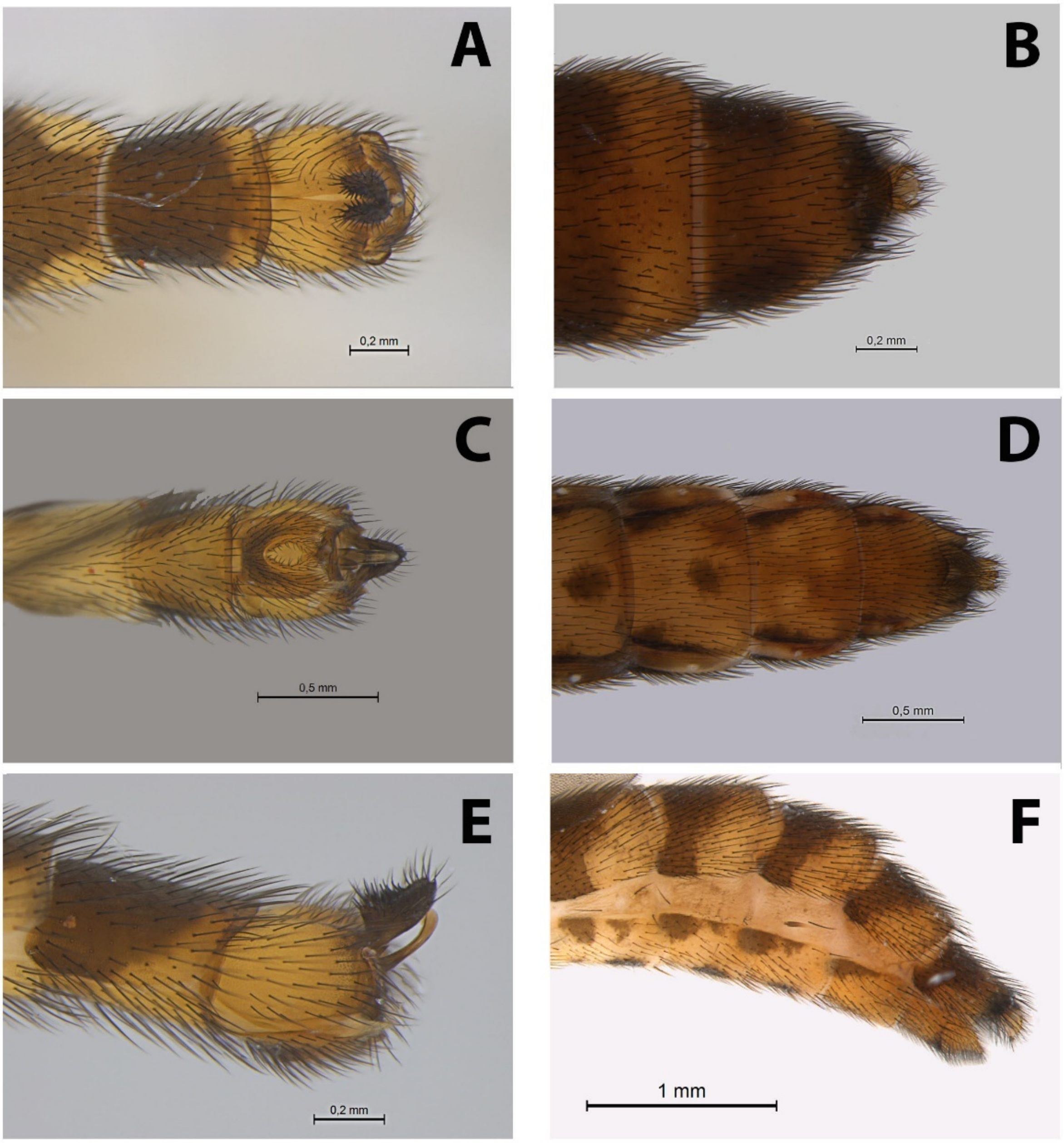

Figure S3. *Neoceroplatus betaryiensis* nov. sp. (**A**) Ventral view of the male terminalia. (**B**) Dorsal view of the female terminalia. (**C**) Dorsal view of the male terminalia. (**D**) Ventral view of the female terminalia. (**E**) Lateral view of the male terminalia. (**F**) Lateral view of the female terminalia.

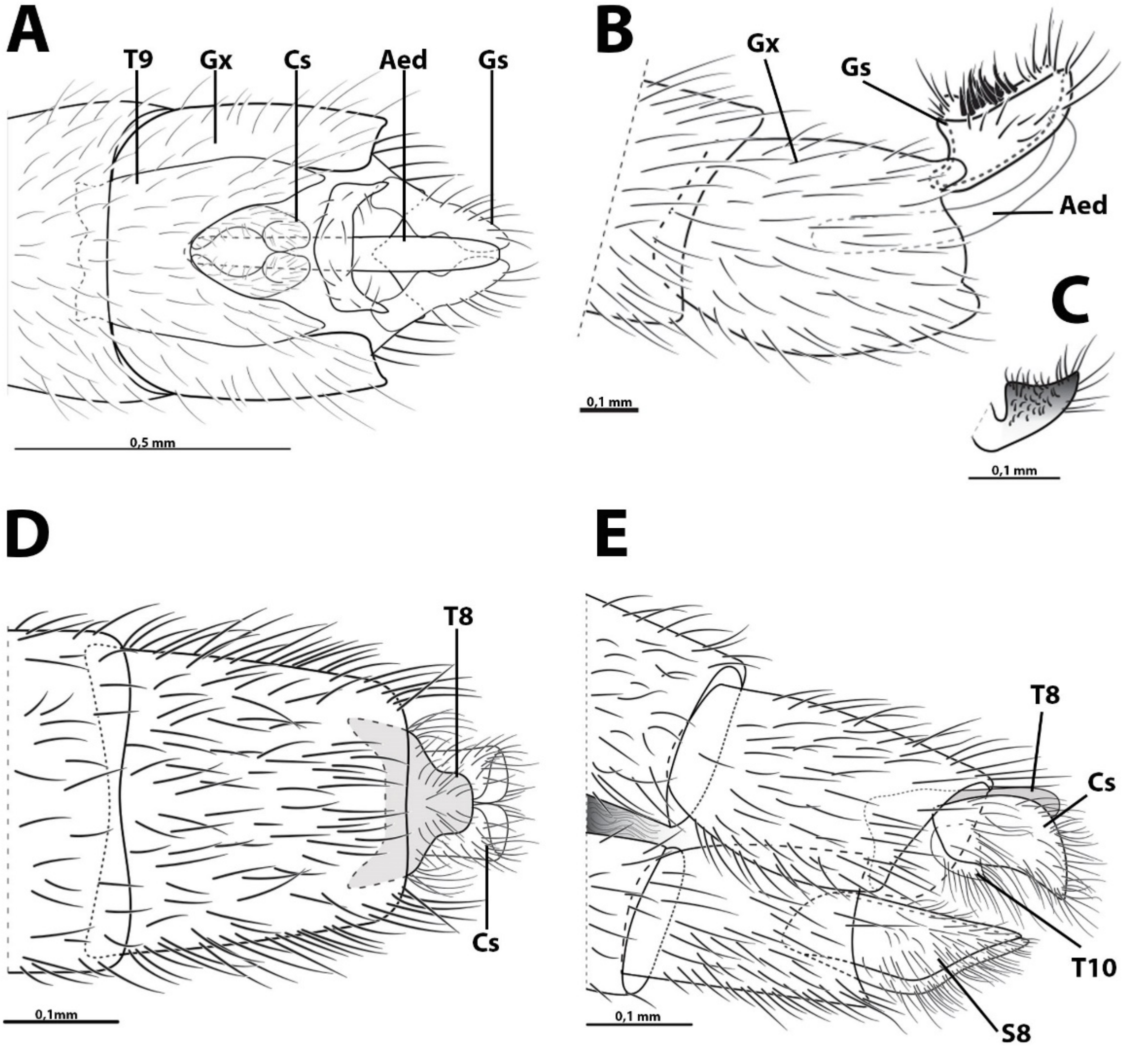

Figure S4. *Neoceroplatus betaryiensis* nov. sp. (**A**) Dorsal view of the male terminalia. (**B**) Lateral view of the male terminalia. (**C**) Detail of the inner face of the gonostylus. (**D**) Dorsal view of the female terminalia. (**E**) Lateral view of the female terminalia. Abbreviations: Aed, aedeagus; Cs, cercus; Gs, gonostylus; Gx, gonocoxite; S8, sternite 8; T10, tergite 10; T8, tergite 8; T9, tergite 9.

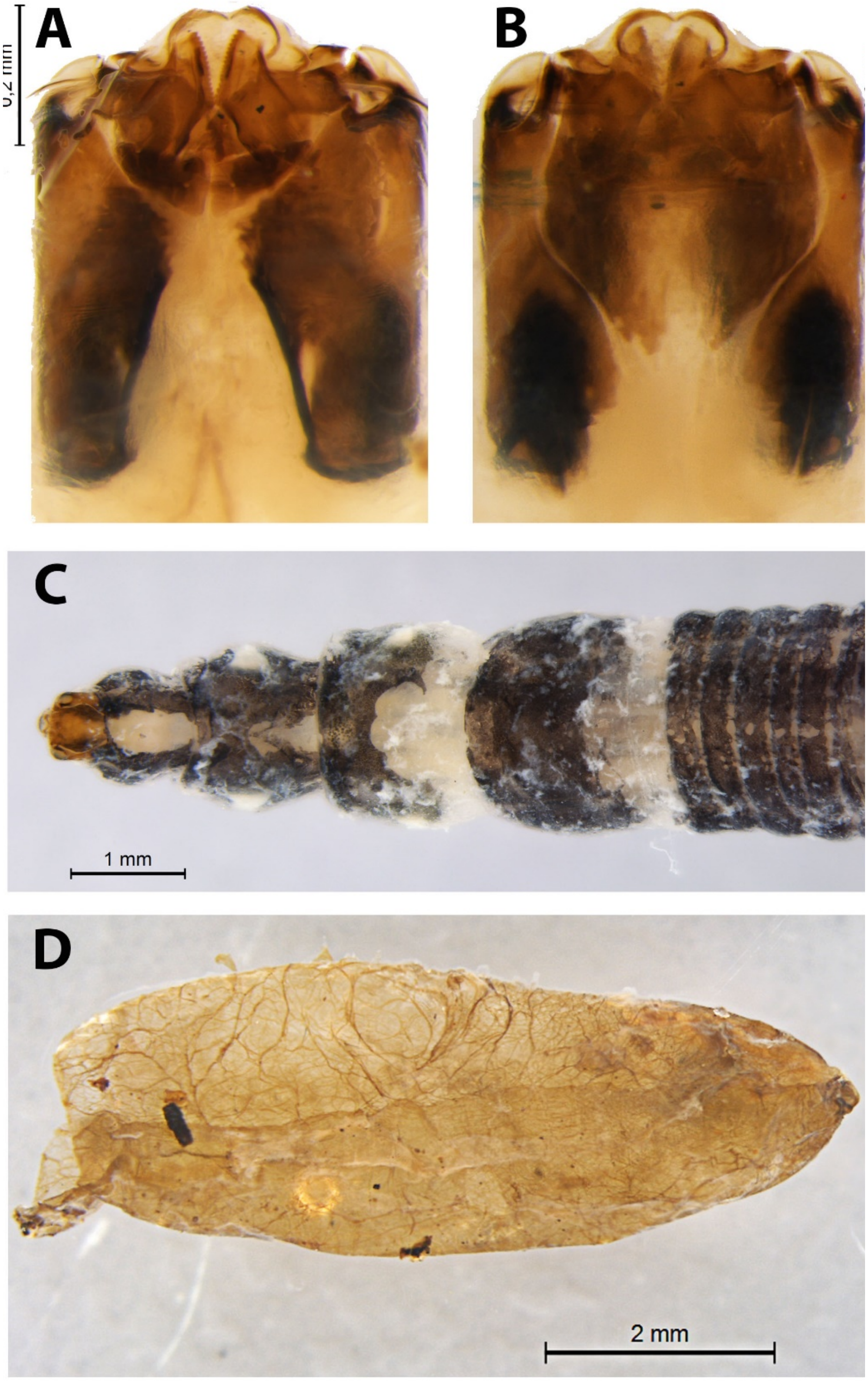

Figure S5. *Neoceroplatus betaryiensis* nov. sp. (**A**) Larva, ventral view of the head. (**B**) Larva, dorsal view of the head. (**C**) Larva, dorsal view of the body (divided into four subquadrate areas). (**D**) Pupa exuvium.
